# Genetic polymorphism of high-molecular-weight glutenin subunit loci in bread wheat varieties in the Pre-Ural steppe zone

**DOI:** 10.18699/VJGB-23-36

**Published:** 2023-07

**Authors:** A.A. Galimova, A.R. Kuluev, K.R. Ismagilov, B.R. Kuluev

**Affiliations:** Institute of Biochemistry and Genetics – Subdivision of the Ufa Federal Research Centre of the Russian Academy of Sciences, Ufa, Russia Federal Research Center the N.I. Vavilov All-Russian Institute of Plant Genetic Resources (VIR), St. Petersburg, Russia; Institute of Biochemistry and Genetics – Subdivision of the Ufa Federal Research Centre of the Russian Academy of Sciences, Ufa, Russia; Bashkir Research Institute of Agriculture of the Ufa Federal Research Centre of the Russian Academy of Sciences, Ufa, Russia; Institute of Biochemistry and Genetics – Subdivision of the Ufa Federal Research Centre of the Russian Academy of Sciences, Ufa, Russia Federal Research Center the N.I. Vavilov All-Russian Institute of Plant Genetic Resources (VIR), St. Petersburg, Russia

**Keywords:** Triticum aestivum, genotyping, baking qualities, high molecular weight glutenins, Glu-1 genes, Triticum aestivum, генотипирование, хлебопекарные качества, высокомолекулярные глютенины, гены Glu-1

## Abstract

High-molecular-weight glutenins play an important role in providing high baking qualities of bread wheat grain. However, breeding bread wheat for this trait is very laborious and, therefore, the genotyping of variety samples according to the allelic composition of high-molecular-weight glutenin genes is of great interest. The aim of the study was to determine the composition of high-molecular-weight glutenin subunits based on the identification of the allelic composition of the Glu-1 genes, as well as to identify the frequency of the Glu-1 alleles in bread wheat cultivars that are in breeding work under the conditions of the Pre-Ural steppe zone (PSZ). We analyzed 26 winter and 22 spring bread wheat varieties from the PSZ and 27 winter and 20 spring varieties from the VIR collection. Genotyping at the Glu-A1 locus showed that the Ax1 subunits are most common in winter varieties, while the predominance of the Ax2* subunits was typical of spring varieties and lines. In the Glu-B1 locus, the predominance of alleles associated with the production of the Bx7 and By9 subunits was revealed for both winter and spring varieties. In the case of the Glu-D1 gene, for all the wheat groups studied, the composition of the Dx5+Dy10 subunits was the most common: in 92.3 % of winter and 68.2 % of spring PSZ accessions and in 80 % of winter and 55 % of spring VIR accessions. The analysis of genotypes showed the presence of 13 different allelic combinations of the Glu-A1, Glu-B1, Glu-D1 genes in the PSZ varieties, and 19 combinations in the VIR varieties. The b b/al/с d allelic combination (Ax2* Вх7+Ву8/8*/9 Dx5+Dy10) turned out to be the most common for the PSZ spring varieties and lines, while for the PSZ winter accessions it was a с d (Ax1 Вх7+By9 Dx5+Dy10); the b с a and b с d genotypes (Ax2* Вх7+Ву9 Dx2+Dy12 and Ax2* Вх7+Ву9 Dx5+Dy10, respectively) occur with equal frequency among the VIR spring accessions; in the group of VIR winter varieties, the combination of the a b/ al d alleles (Ax1 Вх7+Ву8/8* Dx5+Dy10) prevails. The most preferred combination of alleles for baking qualities was found in the spring variety ‘Ekaterina’ and winter varieties ‘Tarasovskaya 97’, ‘Volzhskaya S3’, as well as in lines k-58164, L43510, L43709, L-67, L-83, which are recommended for further breeding programs to improve and preserve baking qualities in the conditions of the Pre-Ural steppe zone.

## Abstract (ru)

Высокомолекулярные глютенины играют важную роль в обеспечении хлебопекарных качеств зерна мягкой пшеницы Triticum aestivum L. Однако селекция мягкой пшеницы по данному признаку весьма трудоемка, поэтому большой интерес представляет генотипирование сортообразцов по аллельному составу генов высокомолекулярных глютенинов. Цель исследования состояла в определении состава субъединиц высокомолекулярных глютенинов на основе выявления аллельного состава генов Glu-1, а также в выявлении частоты встречаемости аллелей генов Glu-1 в сортообразцах мягкой пшеницы, находящихся в селекционной работе в Предуральской степной зоне (ПСЗ). Проанализированы 26 озимых и 22 яровых сортообразца мягкой пшеницы, находящихся в селекционной работе в ПСЗ, и 27 озимых и 20 яровых сортообразцов из коллекции ВИР. Анализ генотипов показал наличие 13 различных аллельных сочетаний генов Glu-A1, Glu-B1, Glu-D1 в сортообразцах ПСЗ и 19 сочетаний – в сортообразцах ВИР. Самым распространенным для яровых сортов и линий ПСЗ оказалось сочетание аллелей b b/al/с d (Ax2* Вх7+Ву8/8*/9 Dx5+Dy10), для озимых – a с d (Ax1 Вх7+Ву9 Dx5+Dy10). Среди яро- вых сортообразцов коллекции ВИР с равной частотой встречались генотипы b с a (Ax2* Вх7+Ву9 Dx2+Dy12) и b с d (Ax2* Вх7+Ву9 Dx5+Dy10); в группе озимых сортов ВИР преобладал генотип a b/al d (Ax1 Вх7+Ву8/8* Dx5+Dy10). Наиболее предпочтительное в целях хлебопечения сочетание аллелей выявлено у ярового сорта Екатерина и озимых сортов Тарасовская 97, Волжская С3, а также у линий к-58164, Л43510, Л43709, Л-67, Л-83, которые рекомендуются для дальнейших селекционных программ по улучшению и сохранению хлебопекарных качеств в условиях Предуральской степной зоны.

## Introduction

The gluten complex of bread wheat grain (Triticum aestivum L.) plays an important role in determining its baking qualities (Gomez et al., 2011). Grain gluten is formed by different groups of proteins (Weiser et al., 2006; Koehle, Weiser, 2013). The most significant among them are high-molecular glutenins: it is between them that intermolecular disulfide bonds resistant to high temperatures are formed. Thus, the number and composition of high molecular weight glutenin subunits (HMW-GS) are important in the formation of bakery product crumb structure, which is formed during baking, and seem to be significant factors that determine the baking quality of wheat grain (Dhaka, Khatkar, 2015).

Each of the loci of high molecular weight glutenins Glu- A1, Glu-B1, Glu-D1 contains two paralogous genes encoding ‘x’- and ‘y’-type proteins, differing in molecular weights and sequences of conservative N-terminal domains (Payne et al., 1982; Shewry et al., 2003). Despite the presence of six Glu-1 genes (coding for HMW-GS Ax1, Bx1, Dx1, Ay1, By1 and Dy1), the number of expressed HMW genes varies from three to five in different varieties of bread wheat, since the genes of the Ax1 and By1 subunits may not be expressed, and expression of the Ay1 subunit is always blocked (Luo et al., 2018). Glutenin genes are characterized by alleles associated with high and low productivity, grain quality, and adaptive potential (Konarev et al., 2000). It was found that good quality of baking, in particular, the elasticity and strength of the dough and the volume of bread, is significantly affected by the subunits encoded by the D subgenome (Yang et al., 2014), followed by the influence of the B subgenome (Zhang L.J. et al., 2015).

Various combinations of the Glu-1 genes alleles of the ABD subgenomes determine the diversity of HMW-GS combinations, which affects the quality of wheat dough and final products. For example, the Glu-A1a allele (encoding the Ax1 subunit) is associated with a high gluten index and a long test development time (Tabiki et al., 2006). The Glu-A1b (Ax2*) allele is usually associated with good dough strength (Vazquez et al., 2012). In turn, the Glu-A1c (null) allele has a negative effect on the quality of the dough (Anjum et al., 2007). The Glu-B1 locus is characterized by the presence of many alleles, among which the Glu-B1f (Bx13+By16) and Glu-B1i (Bx17+By18) alleles have a positive effect on the rheological properties of the dough and baking quality (Guo et al., 2019). Among the alleles of the Glu-B1 locus, a unique allele Glu- B1al was found, which is associated with overexpression of the Bx7 subunit (Bx7OE) and increased dough strength (Zhao et al., 2020). It is known that the presence of the Glu-D1d (Dx5+Dy10) allele contributes to high rheological properties and quality of the dough (Wang G. et al., 1993). If we consider the effect of various HMW-GS on the rheological properties of the dough, they have the following rank order of contribution to the dough strength: Dx5+Dy10 > Dx2+Dy12 > Dx3+Dy12 > Dx4+Dy12 (Payne, Lawrence, 1983; Zhang Y. et al., 2018); at the Glu-B1 and Glu-A1 loci: Bx17+By18 > Bx13+By16 > Bx7+By9 > Bx7+By8 > Bx6+By8 and Ax2* > Ax1 > Null, respectively (Patil et al., 2015).

According to the estimates of the Federal State Budgetary Institution “Centre of Quality Assurance” for 2019, 46.2 % of bread wheat grain in the Republic of Bashkortostan belonged to Class 4, and 20.5 % was not food at all (http://fczerna.ru/). Thus, one of the problems in the cultivation of bread wheat in the Republic of Bashkortostan is the low quality of grain, which depends on the genotype, but the negative impact of the soil and climatic conditions of our region is also not excluded. Based on the importance of HMW-GS in the formation of high baking qualities of wheat grain, elucidating the composition of HMW-GS and their characteristics is an important and relevant task for breeding, aimed, among other things, at improving and preserving the baking qualities of wheat. Such studies have never been carried out before in the conditions of the Pre-Ural steppe zone (PSZ). Therefore, the aims of our work were to determine the allelic state of the Glu-1 loci, to identify the composition of HMW-GS based on PCR, and to analyze the frequency of different genotypes occurrence in bread wheat varieties from the collections of the Bashkir Research Institute of Agriculture of the UFRC of the Russian Academy of Sciences and Federal Research Center the N.I. Vavilov All-Russian Institute of Plant Genetic Resources (VIR)

## Materials and methods

The objects of the study were 48 varieties and lines of bread wheat included in the breeding programs and zoned to the soil and climatic conditions of the PSZ, the baking qualities of which had not been studied, as well as 47 random varieties from the VIR collection with known baking qualities (Suppl. Material 1)1.

Supplementary Materials are available in the online version of the paper: http://vavilov.elpub.ru/jour/manager/files/Suppl_Galimova_Engl_27_4.pdf


Genomic DNA was isolated from dried leaves by a standard method using CTAB (Doyle J.J., Doyle J.L., 1987). Genotyping of the samples was carried out by PCR analysis, visualization of the results was carried out in 1.6 % agarose and 8 % polyacrylamide gels with markers of DNA fragment with lengths of 50 bp and 100 bp (Eurogen, Russia). To identify the allelic states of the Glu-1 genes, we used primer pairs UMN19F/19R (Liu et al., 2008), a/b (Lafiandra et al., 1997), and BxF/ВхR (Ma et al., 2003), as well as ZSBy9aF1/ ZSBy9aR3, ZSBy9aF2/ZSBy9aR2 (Lei et al., 2006) for subgenomes A and B, respectively. In the evaluation of varieties and lines at the Glu-B1 locus, the BxF/BxR primer pair was used to detect alleles of the x-type subunits, and the ZSBy9aF1/ ZSBy9aR3 and ZSBy9aF2/ZSBy9aR2 primer pairs were used to detect alleles of the y-type subunits. The allelic state of the Glu-D1 gene was determined by duplex PCR using primer pairs UMN25F/25R and UMN26F/26R (Liu et al., 2008). Primer sequences and sizes of PCR products are given in Suppl. Material 2. Examples of the electrophoregrams of different alleles of the ABD subgenomes are shown in Suppl. Material 3.

## Results

Glu-A1 locus genotyping

PCR analysis of PSZ winter wheat samples revealed the presence of a allele associated with subunit Ax1 in 21 out of 26 (80.8 %) samples, allele b (Ax2*) – in 4 (15.4 %) and allele c (Ax-null) – in 1 (3.8 %) winter wheat samples. Allele a was found in 3 out of 22 (13.6 %) varieties, allele b was found in 19 (86.4 %) (see Suppl. Material 3, a) varieties in the group of PSZ spring wheat cultivars and lines; cultivars with the c allele were not found in this group

The following results were obtained for samples from the VIR collection: allele a was detected in 21 (77.8 %) winter and 2 (10 %) spring varieties; allele b – in 5 (18.5 %) winter and 13 (65 %) spring varieties; allele c – in 1 (3.7 %) winter and 5 (25 %) spring varieties and lines (see the Table and the Figure, a).

**Table 1.**
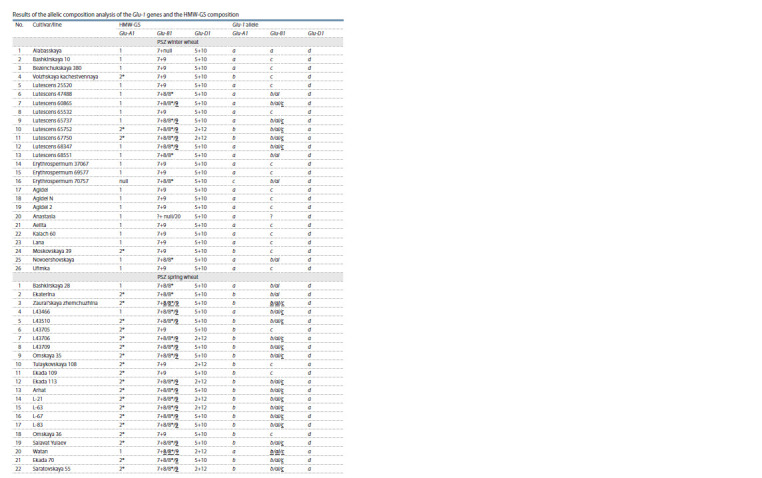
Results of the allelic composition analysis of the Glu-1 genes and the HMW-GS composition

**Tab 1.end:**
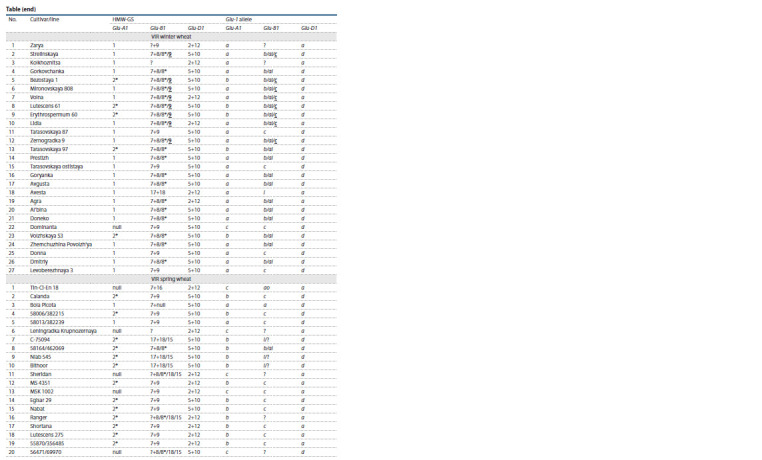
Tab 1.end HMW-GS – high molecular weight glutenin subunits. Bold and underline indicate subunits the expression of which is predominant; alleles and subunits that could not be accurately identified are marked with a question mark

**Fig. 1.**
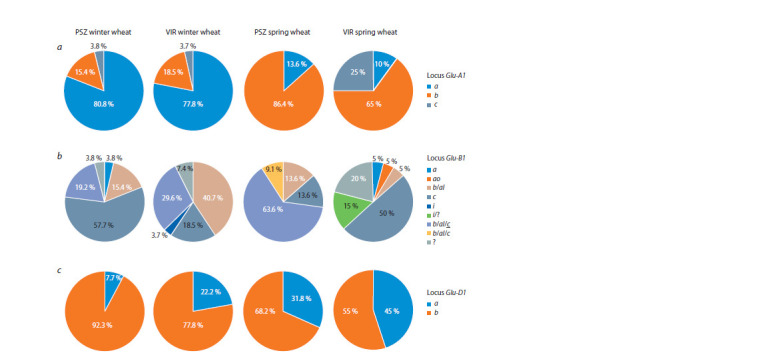
Distribution of the Glu-1 genes alleles in cultivars and lines zoned to the conditions of the PSZ and from the VIR collection. Alleles of ABD subgenomes and their frequency of occurrence: a – subgenome A; b – subgenome B; c – subgenome D.

Glu-B1 locus genotyping

PCR analysis of 26 winter varieties of PSZ showed the presence of the Bx7 subunit in 25 (96 %) varieties (see the Figure, b). In the case of spring cultivars and lines of PSZ, it was revealed that all the studied varieties carry the Bx7 subunit. When analyzing winter varieties of VIR, it was found that all samples carry the allele associated with Bx7 subunits, except for cultivars Zarya, Kolkhoznitsa and Avesta. Amplicons, formed during PCR in the Zarya and Kolkhoznitsa cultivars, have lengths that are not characteristic of loci associated with subunits Bx6, Bx7/7* and Bx17; for cultivar Avesta the Bx17 subunit amplicon was detected (see Suppl. Material 3, c). In the course of genetic analysis of varieties and lines of spring wheat from VIR, the allele encoding the Bx7 subunit was established for 13 cultivars out of 20 (65 %), for 3 cultivars – the Bx17 subunit (15 %), for 4 cultivars it was not possible to identify x-type subunits using the BxF/BxR primers pair (Leningradka Krupnozernaya, Sheridan, Ranger, 56471/69970).

The presence of monomorphic and polymorphic varieties and lines in the studied samples was revealed using pairs of primers ZSBy9aF1/ZSBy9aR3 and ZSBy9aF2/ZSBy9aR2. The share of monomorphic varieties was 80.8 and 70.4 % for winter wheat, and 27.3 and 100 % for spring wheat in the PSZ and VIR samples, respectively. Polymorphic cultivars and lines are the samples, during genotyping of which alleles of several glutenin-coding loci differed in electrophoregrams.

Polymorphism of the Glu-B1 locus is represented by a combination of glutenin subunits Вх7+Ву8/8*/Вх7+Ву9. At the same time, this combination occurs in two forms: the same level of expression of the Ву-type subunits genes (7+8/8*/9) and the predominance of expression of the Ву9 subunit genes (7+8/8*/9). Thus, in the course of genetic analysis using primers ZSBy9aF1/ZSBy9aR3, the allele of the By9 subunit in the monomorphic form was detected in 15 out of 26 (57.7 %) varieties of winter wheat and in 3 out of 22 (13.6 %) varieties of spring wheat of the PSZ samples. The polymorphic form in the combinations of Ву (7+8/8*/9) and Ву (7+8/8*/9) was found in 19.2 and 0 % of winter wheat samples and in 63.6 and 9.1 % of spring wheat samples, respectively. The second pair of primers, ZSBy9aF2/ZSBy9aR2, made it possible to identify the By8/Ву8* allele in 4 winter wheat and 3 spring wheat varieties of PSZ. The Bynull/20 subunit allele was found in the cultivar Anastasia in the selection of winter wheat cultivars and lines of PSZ, while it was not found in the selection of spring wheat varieties of PSZ. The Alabasskaya cultivar was found to carry the a allele, which is characterized by the set of subunits Вх7+Вуnull.

Analysis of varieties and lines of VIR using the ZSBy9aF1/ ZSBy9aR3 primer pair showed that the monomorphic allele of the Ву9 subunit (see Suppl. Material 3, d ) was present in 6 out of 27 (22.2 %) winter wheat and 10 out of 20 (50 %) spring wheat cultivars. Polymorphism in the VIR variety samples is found only in the form of Ву (7+8/8*/9) solely in winter cultivars (29.6 %). It was revealed that 11 out of 27 winter varieties and lines carry the Вх8/8* subunit allele using primers ZSBy9aF2/ZSBy9aR2; the y-type subunit was not established for the Kolkhoznitsa cultivar. It was found that 17 out of 20 spring varieties and lines form 2 reaction products, the three remaining varieties have 0, 1 and 3 amplification reaction products with primers ZSBy9aF2/ZSBy9aR2. Thus, according to the results of PCR of two primer pairs ZSBy9aF1/ ZSBy9aR3 and ZSBy9aF2/ZSBy9aR2, it can be seen that 10 out of 20 (50 %) spring cultivars carry the allele associated with the production of the Ву9 subunit, 7 (35 %) cultivars carry the 8/8*/18/15 subunit allele, 1 cultivar (Bola Picota) – the null/20 subunit allele, 1 cultivar (Tin-Ci-En 18) – the By16 subunit allele (see the Table and the Figure, b).

Glu-D1 locus genotyping

Identification of the allelic composition of the Glu-D1 gene was carried out using two pairs of primers to simultaneously detect alleles of genes encoding subunits Dх5 and Dу10 (5+10), Dх2 and Dу12 (2+12). When using these primers for samples with subunits 5+10 we observed the presence of 397 and 281 bp amplicons (allele d), and for samples with subunits 2+12 – 415 and 299 bp amplicons (allele a) (see Suppl. Material 3, b). Thus, in the sample of PSZ cultivars, the d allele was detected in 24 (92.31 %) winter and 15 (68.18 %) spring cultivars; in a sample of varieties and lines from the VIR collection, this allele was found in 24 (88.9 %) winter and 11 (55 %) spring cultivars of wheat (see the Table and the Figure, c).

Glu-1 gene allele combination (ABD)

The presence of 8 different combinations of the Glu-1 (ABD) gene alleles was characteristic for winter wheat, and 9 different combinations of alleles – for spring wheat of PSZ varieties. The predominant alleles combination for winter wheat was the combination of alleles а с d – it was detected in 13 out of 26 varieties (50 %) of the samples of PSZ, while in the case of spring wheat, the combination of b b/al/с d was predominant (36.4 %), so a significant number of spring varieties had a genotype with b b/al/с а alleles combination (22.7 %).

The presence of 10 different combinations of the Glu-1 (ABD) gene alleles was revealed in 27 winter wheat varieties and 11 combinations of alleles – in the spring wheat forms from 20 VIR cultivars and lines. The predominant combinations of alleles of winter varieties turned out to be the combination a b/al d (29.6 %; 8 varieties out of 27), in spring varieties, combinations of alleles b с d and b с a were the most common (20 % of each combination; 4 varieties out of 20). In addition, the combination of alleles b i/? d was observed in 15 % of varieties (3 varieties out of 20).

## Discussion

To date, the existence of correlations between the presence of certain alleles of the Glu-1 genes and indicators of the baking quality of bread wheat grain has been shown. HMW-GS genes loci are characterized by high polymorphism (Patil et al., 2015), which may be one of the reasons for the genetic variability of wheat cultivars in terms of the rheological and technological properties of the dough. On the other hand, these qualities are rather difficult to control in classical breeding. Therefore, genotyping of HMW-GS alleles is an important task aimed at the effective selection of parental forms with high baking qualities, and the results obtained can be applied in marker-assisted and genomic selection of bread wheat. However, first it is necessary to carry out work on the assessment of the genetic diversity of bread wheat cultivars according to the allelic composition of high molecular weight glutenin genes in certain regions, which was the subject of our study.

The Ax1 and Ax2* subunits have a positive effect on the quality of the dough, while the null subunit has a negative effect (Anjum et al., 2007). We have found that in winter varieties for subgenome A, allele a (Ax1) is predominant, while in spring varieties it is allele b (Ax2*) (see the Figure, a). This pattern is typical for both studied groups (PSZ and VIR).

Eight different Glu-B1 alleles were identified for all the studied varieties and lines, the total number of which was 95 (see the Figure, b). At the same time, it was not possible to identify the exact allele for 7 varieties, since reaction products formed during PCR either did not correspond in size to the expected ones, or were absent. These samples require further research, including by sequencing, for the possible carriage of new alleles. Among the eight identified alleles, the highest frequency of occurrence was determined for the c allele (Вх7+Ву9) (34.7 % of the total number of all PSZ and VIR samples), which correlates with the results of other studies that indicate a wide distribution of this allele (Payne, Lawrence, 1983; Gianibelli et al., 2001).

When analyzing a sample of varieties and lines of bread wheat PSZ, it was found that the most common alleles are c and b/al/c. Thus, 57.7 % of winter varieties of PSZ carry the c allele (Вх7+Ву9), 63.6 % of spring varieties and lines of PSZ carry the b/al/c allele (Вх7+Ву8/8*/9). However, when analyzing varieties and lines from the VIR collection, it was revealed that most of the c alleles are carried by spring wheat varieties (50 %), and winter wheat varieties with a frequency of 40.7 % carry the b/al allele (Вх7+Ву8/8*). Thus, the winter wheat varieties of PSZ were more often monomorphic, in contrast to the spring wheat varieties, for which the production of PCR products characteristic of both the b/al allele (Вх7+Ву8/8*) and the c allele (Bx7+By9) was detected. At the same time, it should be noted that the production of amplicons associated with the c allele occurs much more often compared to the amplicons of the b/al allele (varieties have the b/al/c genotype (Вх7+Ву8/8*/9)). The distribution of alleles b/al (Вх7+Ву8/8*) and c is not surprising, since these alleles are associated with good baking qualities (Payne, Law- rence, 1983).

In the studied samples, it is worth paying attention to the winter wheat cultivar Avesta from the VIR collection, since this cultivar is likely to carry the Glu-B1i allele (Вх17+Ву18) associated with high grain quality, and can be selected for breeding programs aimed at improving grain quality. In addition to the Avesta cultivar, it is necessary to carry out additional studies in order to establish the exact allele, namely, to identify the allele of the y-type subunit in spring wheat varieties from VIR – C-75094, Niab 545, Bithoor. If the presence of the Glu-B1i allele (Вх17+Ву18) is confirmed, these wheat varieties can also be used in breeding programs. When planning hybridization work, it should be borne in mind that the Alabasskaya and Bola Picota cultivars carry the a allele (Bx7) of the B subgenome, which is associated with a rather low grain quality assessment. The Bola Picota cultivar, according to VIR data, really has low baking qualities (see Suppl. Material 1). So, the analysis of the Glu-Bx locus showed that the carriage of the allele associated with the production of Bx7 subunits is predominant for both winter and spring forms of bread wheat. Genetic analysis of the Glu-By locus revealed the greatest distribution of the allele associated with the production of the By9 subunit in wheats of both forms of vernalization.

During the genetic analysis of the Glu-D1 gene, it was revealed that all the studied varieties and lines of PSZ and VIR carry one of two alleles – a or d. The results of studies of VIR samples also indicate a wide distribution of these alleles and the Dx2+Dy12 and Dx5+Dy10 glutenin subunits encoded by them (Ayala et al., 2016). In addition, it was found that for all the studied wheat groups, the composition of the subunits Dx5+Dy10 (allele d) is the most common (frequency of occurrence in total in cultivars and lines of PSZ is 83.3 %; in the total sample of VIR ‒ 72.3 %). At the same time, the frequency of occurrence of this allele in winter wheat varieties is higher than in spring ones (see the Figure, c). It is known that the d allele (Dx5+Dy10) of the Glu-D1 locus has a pronounced positive effect on flour quality (Payne, Lawrence, 1983), which is consistent with selection aimed at improving the baking qualities of grain. The second allele of the D subgenome identified by us is the allele a (Dx2+Dy12); theoretically, it can have a negative impact on the production of high-quality pan bread, but it is recommended for cultivars used for making hearth bread and noodles. However, it is worth mentioning that this allele is not always associated with poor grain quality. Relatively recently, genes have been discovered that cause the synthesis of Dy12.7 subunits (Peng et al., 2015) and Dy12** (Du et al., 2019), which have similar molecular weights to standard Dy12, but are associated with increased grain quality.

In addition to the influence of single alleles on the quality of the final product, the quality of dough and bread is affected by the total effect of alleles of all three subgenomes (Payne et al., 1981; Wang Z.J. et al., 2018; Zhao et al., 2020). In the sample of all analyzed wheat samples, 24 allelic combinations were identified at the Glu-1 locus, among which the combination of alleles b b/al d (Ax2* Bx7+By8/8* Dx5+Dy10) is the most preferable for baking purposes (Pirozi et al., 2008). A similar combination of alleles was detected in the spring wheat cultivar Ekaterina. According to the data given in the Russian State Register for Selection Achievements, this cultivar has good baking qualities. Among the varieties of VIR, the combination of alleles b b/al d (Ax2* Bx7+By8/8* Dx5+Dy10) is found in winter wheat cultivars Tarasovskaya 97, Volzhskaya S3 and spring wheat line k-58164. Cultivars Tarasovskaya 97 and Volzhskaya S3, according to the Russian State Register for Selection Achievements, have good baking qualities. Thus, winter wheat cultivars Tarasovskaya 97, Volzhskaya S3 and spring wheat cultivar Ekaterina can be taken into account when selecting parental pairs for breeding bread wheat in order to improve baking qualities

Combinations of alleles a с d (Ax1 Вх7+Ву9 Dx5+Dy10) (50 % – PSZ, 14.8 % – VIR) and a b/al d (Ax Вх7+Ву8/8* Dx5+Dy10) (11.5 % – PSZ, 29.6 % – VIR) turned out to be the most common in the total sample of winter wheat varieties and lines. It was reported that these combinations of subunits are rated 9 and 10 scores on the Payne scale. From the frequency of occurrence of alleles a с d (Ax1 Вх7+Ву9 Dx5+Dy10) (9 scores on the Payne scale) and a b/al d (Ax1 Вх7+Ву8/8* Dx5+Dy10) (10 scores) in the sample of winter wheat varieties of PSZ and the VIR collection, it can be seen that in regions with climatic conditions of PSZ, selection went towards the fixation of the с allele of the subgenome B in comparison with the VIR samples, which collected varieties and lines from different regions and countries (see the Figure, b). However, in the studied sample of spring wheat varieties, the opposite picture is observed – classical selection in PSZ goes towards the acquisition of the b allele (Bx7+By8) of the B subgenome (see the Figure, b). So, according to the frequency of occurrence of genotypes b b/al/c a (Aх2* Bх7+Ву8/8*/9 Dх2+Dу12) (22.7 % – PSZ, 0 % – VIR), b b/al/c d (Aх2* Bх7+Ву8/8*/9 Dх5+Dу10) (36.4 % – PSZ, 0 % – VIR), b c d (Aх2* Bх7+Ву9 Dх5+Dу10) (13.6 % – PSZ, 20 % – VIR) and b c a (Aх2* Bх7+Ву9 Dх2+Dу12) (0 % – PSZ, 20 % – VIR), it can be seen that heteromorphism of the Glu-B1 locus appeared in the group of PSZ varieties and lines.

The lowest HMW-GS scores according to the Payne scale have genotypes b c a (Aх2* Bх7+Ву9 Dх2+Dу12) (Glu-1 score = 7). This combination of alleles was identified in the spring wheat cultivar Tulaykovskaya 108, but according to the Russian State Register for Selection Achievements, this cultivar belongs to strong wheat with good baking qualities. However, according to our data, in the conditions of the Republic of Bashkortostan, cultivar Tulaykovskaya 108 is characterized only by satisfactory baking qualities. These contradictory data, among other things, are probably related to the climatic conditions of the regions, and the genotype of the cultivar may become a limiting factor under adverse external conditions. In the group of cultivars and lines from the VIR collection, genotype b c a was found in 20 % of spring wheat samples. In addition to the b c a genotype, combinations of alleles b b/ al/c a (Aх2* Bх7+Ву8/8*/9 Dх2+Dу12) (Glu-1 score = 7 or 5) and c с a (Ax-null Bх7+Ву9 Dх2+Dу12) (Glu-1 score = 5) have low scores on the Payne scale. The last combination of alleles has been found in one spring wheat line of VIR – MSK 1002. Genotype b b/al/c a (Aх2* Bх7+Ву8/8*/9 Dх2+Dу12), despite the low Glu-1 score, is widespread in the group of spring wheat varieties PSZ (22.7 %), two winter lines of PSZ also have this genotype – Lutescens 65752 and Lutescens 67750.

## Conclusion

Polymorphism of bread wheat cultivars zoned to the conditions of PSZ was studied for the genes of high molecular glutenins Glu-1 by PCR analysis. Among the 26 studied winter cultivars of PSZ, the predominant majority had genotypes with the a c d allele (Ax1 Вх7+Ву9 Dx5+Dy10). The b b/ al/c d (Aх2* Bх7+Ву8/8*/9 Dх5+Dу10) alleles combination was found to be dominant for 22 spring cultivars of PSZ. Among the 24 identified ones, the combination of alleles b b/al d (Ax2* Bx7+By8/8* Dx5+Dy10) of the Glu-1 genes is the most preferred for baking purposes. This genotype was detected in the spring wheat cultivar Ekaterina and winter wheat cultivars Tarasovskaya 97, Volzhskaya S3 and spring wheat line k-58164. Of the promising spring wheat lines of the PSZ, the lines L43510, L43709, L-67, L-83, which are recommended for further breeding programs to improve and preserve baking qualities in the conditions of the Pre-Ural steppe zone, may have the highest score according to Payne.

## Conflict of interest

The authors declare no conflict of interest.
